# Effect of Female Body Mass Index on Oocyte Quantity in Fertility Treatments (IVF): Treatment Cycle Number Is a Possible Effect Modifier. A Register-Based Cohort Study

**DOI:** 10.1371/journal.pone.0163393

**Published:** 2016-09-21

**Authors:** Mette Wulf Christensen, Hans Jakob Ingerslev, Birte Degn, Ulrik Schiøler Kesmodel

**Affiliations:** 1 Aarhus University Hospital, Aarhus, Denmark; 2 Department of Clinical Medicine, Aarhus University, Aarhus, Denmark; Medical University of Vienna, AUSTRIA

## Abstract

**Introduction:**

Overweight and obese women may require higher doses of gonadotrophin when undergoing In Vitro Fertilization Treatment (IVF). Consequently, one may expect a sub-optimal oocyte retrieval in the first treatment cycle and thus a larger compensation in gonadotrophin-dose in the following treatment-cycles and a more favorable outcome. The main objective was to explore if treatment cycle number modifies the outcome when investigating the effect of female Body Mass Index (BMI) on oocyte quantity in IVF.

**Material and Methods:**

A historical cohort study was conducted on 5,342 treatment-cycles during the period 1999–2009. Exclusion criteria were missing information on BMI or treatment type. Further, women were excluded if they had ovulated before oocyte retrieval. According to baseline BMI, women were divided into four categories following the World Health Organization standards. Multiple linear regressions analyses were performed accounting for the non-independence of ≥2 cycles in a woman.

**Results:**

Stratification according to cycle number revealed a more suboptimal outcome in the first treatment- cycles than in the following cycles, suggesting a possible interaction or effect modification from cycle number or a factor related to cycle number. The median dose of total follicular stimulating hormone given to the four BMI groups could not straight forwardly explain the less optimal oocyte outcome observed in first treatment cycles. No statistically significant differences were observed in oocyte yield for underweight, overweight and obesity compared to normal weight women when analyzing all treatment-cycles. Overweight women had significantly fewer mature (MII) oocytes (*p* = 0.009) than normal weight women, whereas no differences was observed for underweight and obese women.

**Conclusion:**

Our study suggests a possible interaction or effect modification related to treatment cycle number. Investigating the effects of BMI on IVF-results in first treatment-cycles alone should be carried out cautiously.

## Introduction

Overweight and obesity is an increasing health problem throughout the world, and obesity has gradually become a World-wide epidemic [1]. In many European countries, including Denmark, nearly 30% of the women in the reproductive age are either overweight or obese [2–4]. Several studies have reported significant associations between obesity and the reproductive system, including reduced fecundity and a higher risk of suffering from menstrual disorders and anovulation [5,6]. Hence, obese women have a 3 fold higher risk of infertility due to anovulation when compared to normal weight women [6]. This has led to more overweight and obese women seeking help from assisted reproductive technologies (ART) to achieve a pregnancy.

In ART, the ovaries are stimulated with follicular stimulating hormone (FSH) to obtain growth of multiple follicles, from which oocytes are retrieved and fertilized in vitro. Hormonal changes due to obesity and the increased risk of insulin resistance are suggested to inhibit follicular development, thereby reducing the number of oocytes retrieved [7–10]. The oocyte remains arrested in the germinal vesicle stage (prophase 1 of meiosis) from oogenesis through follicle formation and growth. The maturation does not continue until the preovulatory GnRH and LH surge where the oocyte reaches the metaphase II of meiosis and is now ready for fertilization and completion of maturation [11]. Also the maturity of oocytes is suggested to be inhibited in obese women due to hormonal changes, reducing the chance of achieving a pregnancy by ART [10,12,13]. Consequently, it is of great interest to understand how overweight and obesity affects the oocyte outcome in ART.

The literature regarding the association between obesity and oocyte outcome is, however, not clear [10,14,15]. The inconsistent findings could somehow be explained by lack of consistency in the methods used, insufficient adjustment for potential confounders or different definitions of BMI categories and outcome measures [3,16]. Another point to consider is that some studies report on several treatment-cycles per women, whereas others only report on first treatment-cycles in order to avoid analyses of multiple, non-independent cycles in the same women [7,17–20]. However, studies have demonstrated that overweight and obese patients require higher doses of gonadotrophin to achieve optimal ovarian stimulation compared to their normal-weight counterparts [7,9,17,18,21]. Due to the risk of ovarian hyperstimulation syndrome (OHSS), insufficient gonadotrophin stimulation may be used in first cycles leading to sub-optimal oocyte retrieval. As a consequence in the following treatment cycles one may expect a larger compensatory gonadotrophin dose to be used, and hence a more favorable outcome.

Considering the possible influence of gonadotrophin dose and the inconsistent findings in the literature regarding the impact of overweight and obesity on oocyte outcome, the objective of this study was twofold: 1) To explore whether there was a difference between the number of oocytes and the number of mature oocytes when stratifying on cycle number and 2) to evaluate the effect of BMI on the oocyte outcome on all treatment-cycles during a 10 year period at the Fertility Clinic, Aarhus University Hospital, Denmark.

## Materials and Methods

### Study Population

**Fig 1:**
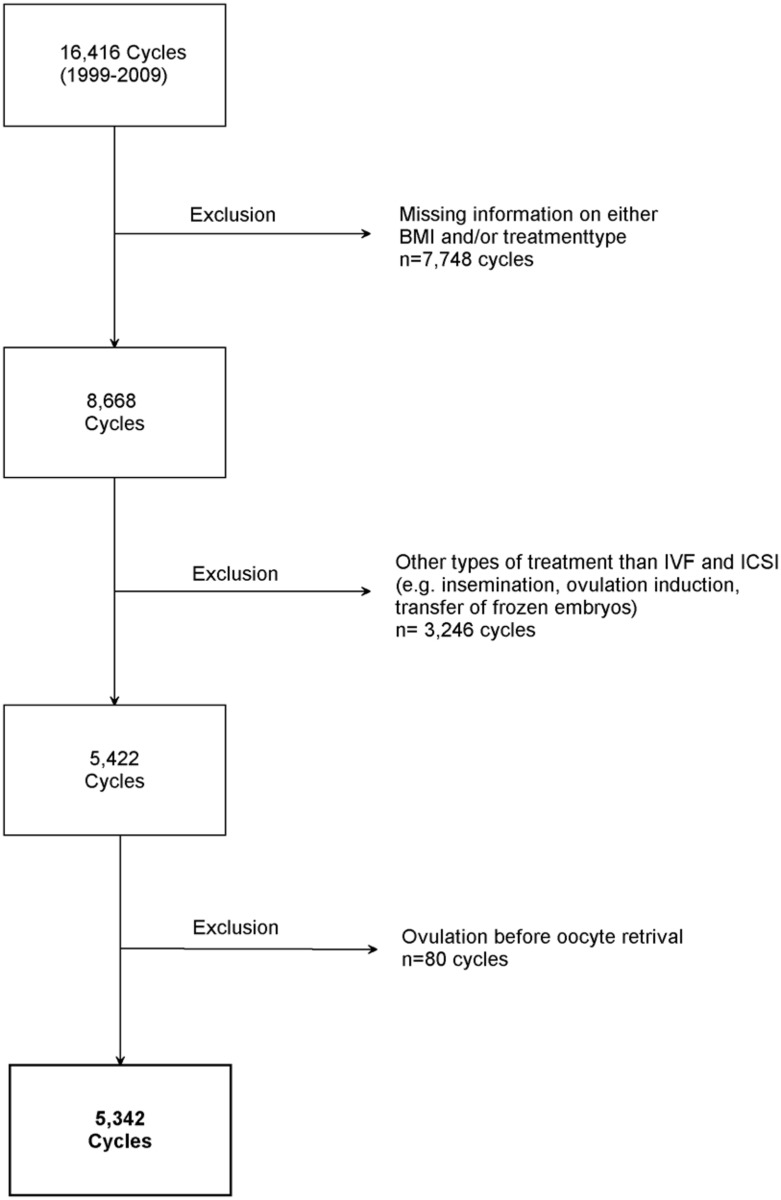
Flowchart. Inclusion and exclusion of treatment-cycles to the study.

We conducted a historical cohort study on all ART cycles (*n* = 16 416), performed at the Fertility Clinic, Aarhus University Hospital, during the period 1999–2009. Women were included in the study if they were receiving treatment with either In Vitro Fertilization (IVF) or Intra Cytoplasmic Sperm Injection (ICSI), and if they had a BMI recorded in their electronic medical chart (*n* = 5422). Women were excluded if they were receiving other types of treatment, for example insemination (IUI) (*n* = 3246) or had missing information on either BMI and/or treatment type (*n* = 7748). Further, 80 women were excluded due to ovulation before oocyte retrieval. A total of 5342 IVF and ICSI treatment-cycles underwent statistical analysis (Fig 1).

The majority of the women (> 90%) underwent controlled ovarian stimulation with recombinant FSH (rFSH) and a GnRH agonist. Follicle development was monitored with the use of ultrasound and ovulation was induced by hCG-administration (Pregnyl^®^) when observing three or more follicles ≥ 17 mm. Oocyte retrieval was performed transvaginally ultrasonography-guided approximately 34–36 hours after the administration of hCG.

Embryologists determined the total number of oocytes retrieved per cycle and evaluated the maturity of ICSI oocytes. In ICSI-cycles cumulus cells were removed 2–3 hrs. after retrieval. Only metaphase II oocytes (MII) were considered mature. For IVF cycles, maturity was not determined systematically and was not used for statistical analysis.

### Data Set

Data for this study were collected from the electronic medical database,, located at the Fertility Clinic, Aarhus University Hospital. The data consisted of clinical information typed in the database in connection with each treatment-cycle and data obtained from a questionnaire given to the women just before the beginning of their first treatment-cycle. The questionnaire contained information regarding the women’s smoking habits, alcohol consumption, coffee consumption, and height and weight. Both the answers from the questionnaire and the self-reported height and weight were typed into the database in connection with the first treatment-cycle.

The BMI was defined as the weight in kilograms divided by the square of the height in meters (kg/m^2^). We used the World Health Organization (WHO) classification of BMI categories to divide the patient population into four groups: < 18.5 kg/m^2^ (underweight), 18.5–24.9 kg/m^2^ (normal), 25.0–29.9 kg/m^2^ (overweight), > 30.0 kg/m^2^ (obese).

### Statistical Analysis

The statistical analyses were carried out using STATA, statistical software intercooled version 11.2 (Stata Corporation, College Station, Texas USA). Continuous variables are presented as medians and categorical variables as frequencies and percentages.

Multiple linear regression analyses were used. We calculated robust standard errors in order to account for the non-independence of ≥2 cycles within the same woman. The two outcome measures were logarithmically transformed prior to the analyses to normalize the distribution of the residuals. The analyses were further stratified according to cycle number, defined as, 1) first treatment- cycle, and 2) second treatment-cycle and onwards (2^nd+^). Based on the literature potential confounders were selected [3,22–25]. We adjusted for the following potential confounders: age (continuous), smoking (categorical:0 cigarettes/day; 0–10 cigarettes/day;>11 cigarettes/day), alcohol (categorical: no alcohol; 1–5 units/day; 6–10 units/day; > 10 units/day), coffee (categorical: no coffee; 1–5 cups/day; ≥ 6 cups/day), infertility diagnosis (categorical: anovulation; tuba factor; male factor; unexplained; other), day 3 serum-FSH level (continuous) and total gonadotrophin (FSH) dose (continuous). In order to account for an uneven distribution of women included per year during the 10 year study period, sub-analyses adjusting for calendar year were performed. Furthermore, a possible interaction between smoking and BMI and between age and BMI were investigated. It was expected that missing values could occur for some of the explanatory variables used in the statistical analyses. For categorical variables, the missing values were included in the analyses as an independent category. For continuous variables, an estimate of the average for each BMI category was calculated on the basis of the available values. Medians (25^th^/75^th^ percentile) were used for non-normally distributed data, while means (±SD) were used for normally distributed data. A post- hoc analysis was performed investigating the clinical pregnancy rate in the four BMI groups stratified on treatment cycle number. Clinical pregnancy rate was defined as the presence of minimum 1 gestational sac 5 weeks after transfer of 1 or 2 embryos divided by the total number of cycles started. Multiple logistic regression analyses were used, adjusted for the same potential confounders as in the linear regression model. Robust standard errors were calculated. A p-value of 0.05 was considered statistically significant. The study was approved by the Danish Data Protection Agency (jr.no 1-16-02-172).

## Results

A total of 3251 IVF and 2091 ICSI cycles were analyzed. The underweight women accounted for 3% of the study population, whereas overweight and obese accounted for 21.9 and 8.9%, respectively (Table 1).The median age was a little lower in the underweight category (30.0 years) compared to the other BMI categories (32.0 years).

**Table 1:** Clinical Baseline Characteristics per Cycle and per Woman According to BMI. Data is presented as median (25/75 percentile) unless otherwise stated.

	Underweight	Normal weight	Overweight	Obese
	*(< 18*.*5 kg/m* ^*2*^*)*	*(18*.*5–24*.*9 5 kg/m*^*2*^*)*	*(25*.*0–29*.*9 kg/m* ^*2*^*)*	*(≥ 30 kg/m* ^*2*^*)*
**Number of cycles n (%)**	158 (3.0)	3539 (66.3)	1171 (21.9)	474 (8.9)
**Age (yrs)**^a^	30(28/33)	32 (29/35)	32(29/35)	32(28/35)
**Weight (kg)**^a^	51 (48/53)	61 (57/65)	77(72/81)	91(87/100)
**BMI (kg/m**^**2**^**)**	18.0(17.5/18.3)	21.6 (20.4/23.0)	27.0 (25.9/28.1)	31.9(30.8/34.5)
**Primary cause of infertility, n (%)**				
*Anovulation*	10 (6.3)	117 (3.3)	78 (6.7)	48 (10.1)
*Tuba factor*	34 (21.5)	721 (20.4)	286 (24.4)	103 (21.7)
*Male factor*	52 (32.9)	1340 (37.9)	457 (39.0)	194 (40.9)
*Unexplained*	40 (25.3)	853 (24.1)	193 (16.5)	85 (17.9)
*Other*	21 (13.3)	486 (13.7)	153 (13.1)	44 (9.3)
*Data missing*	1 (0.6)	22 (0.6)	4 (0.3)	0 (0)
**Primary cause of infertility, ICSI cycles only, n (%)**				
*Anovulation*	2 (3.1)	10 (0.8)	13 (2.6)	13 (5.9)
*Tuba factor*	3 (4.6)	63 (4.8)	32 (6.4)	13 (5.9)
*Male factor*	42 (64.6)	1006 (77.2)	358 (71.0)	160 (73.1)
*Unexplained*	5 (7.7)	69 (5.3)	27 (5.4)	11 (5.0)
*Other*	12 (18.5)	146 (11.2)	71 (14.0)	22 (10.1)
*Data missing*	1 (1.5)	9 (0.7)	3 (0.6)	0 (0)
**Coffee, n (%)**				
*No coffee*	81 (51.3)	1140 (32.2)	407 (34.8)	208 (43.9)
*1–5 cups/day*	55 (34.8)	1958 (55.3)	594 (50.7)	204 (43.0)
*> 5 cups/day*	12 (7.6)	317 (9.0)	115 (9.8)	33 (6.7)
*Data missing*	10 (6.3)	124 (3.5)	55 (4.7)	29 (6.1)
**Smoking, n (%)**				
*No smoking*	104 (65.8)	2356 (71.7)	775 (66.2)	326 (68.8)
*0–10 cigarettes/day*	36 (22.8)	572 (16.3)	169 (14.4)	77 (16.3)
*> 10 cigarettes/day*	12 (7.6)	535 (15.1)	199 (17.0)	59 (12.3)
*Data missing*	6 (3.8)	76 (2.2)	28 (2.4)	12 (2.5)
**Alcohol, n (%)**				
*No alcohol*	129 (81.6)	2715 (76.7)	970 (82.2)	442 (93.2)
*1–5 units/day*	25 (15.8)	753 (21.3)	177 (15.1)	24 (5.1)
*6–10 units/day*	0 (0)	3 (0.1)	0 (0)	0 (0)
*> 10 units/day*	0 (0)	0 (0)	0 (0)	0 (0)
*Data missing*	4 (2.5)	68 (1.9)	24 (2.1)	7 (1.5)
**Fertilization method, n (%)**				
*IVF*	93 (58.9)	2236 (63.2)	667 (57.0)	255 (53.8)
*ICSI*	65 (41.1)	1303 (36.8)	504 (43.0)	219 (46.2)
**Oocytes**	6 (4/11)	6 (4/10)	6 (4/10)	6 (4/10)
**MII oocytes**^b^	6 (3/12)	6 (3/9)	5 (2/8)	5 (2/8)
**Basline FSH (IU)**	6.1 (4.7/7.3)	6.6 (5.2/7.5)	5.9 (4.7/6.9)	5.9 (4.7/7.1)
**Total FSH dose (IU)**	1572 (1000/1950)	1650 (1200/2025)	1800 (1350/2200)	2000 (1550/2600)
**Clinical pregnancy rate, n (%)**	44 (27.9)	905 (25.6)	247 (21.1)	106 (22.4)

^*a*^ due to settings in the clinical database age and weight are only available as whole numbers

^*b*^ only ICSI-cycles

Table 1 presents the clinical characteristics of the study participants according to BMI categories. The most common reason for infertility was male factor in all four BMI categories. Obese women were more likely to suffer from anovulation compared to normal weight women (10.1% versus 3.3%). Overweight and obese women received higher total FSH-dose than normal weight women.

### Number of Oocytes

**Fig 2:**
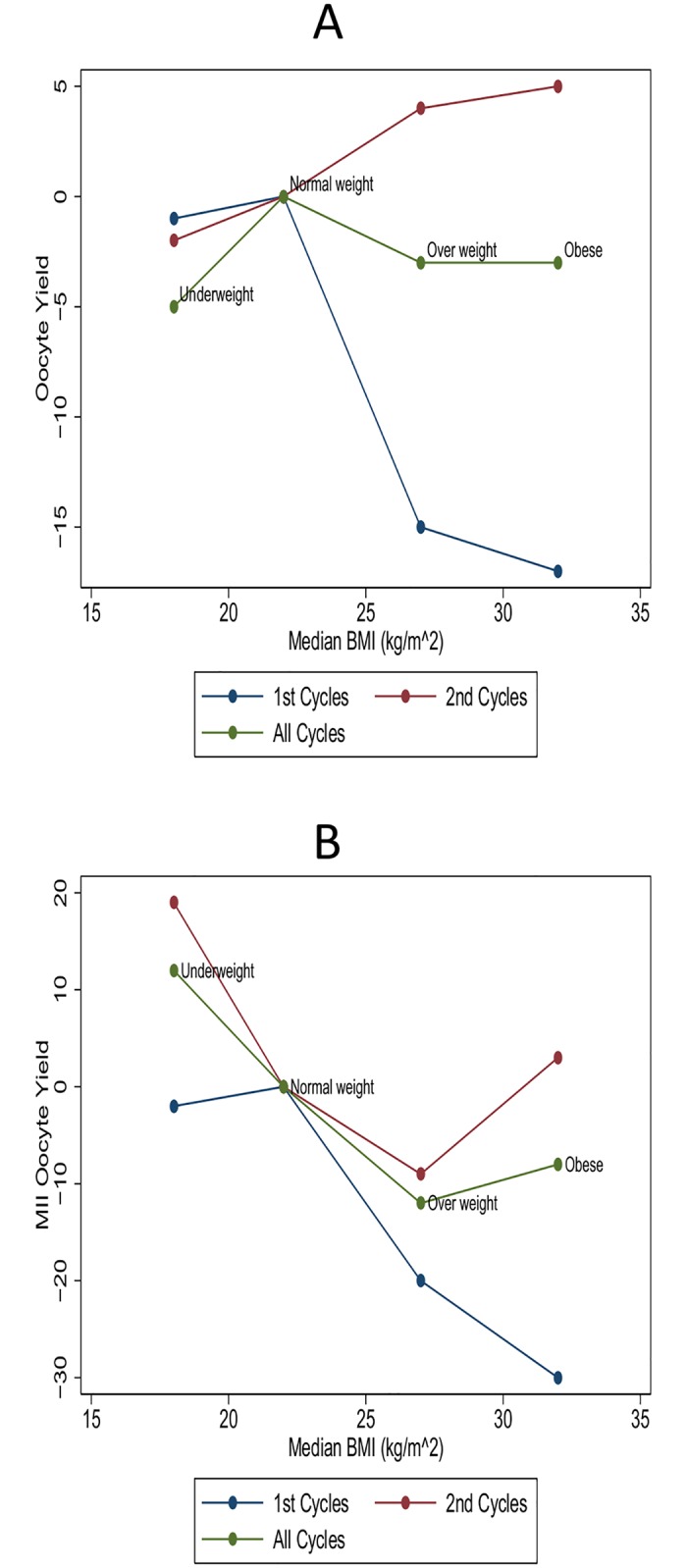
Number of Oocytes and MII Oocytes According to BMI and Cycle Number. (A) Number of oocytes according to median BMI for each BMI group and cycle number. Estimates are shown as back transformed multiple linear regression estimates and each estimate shows the percentage of oocytes retrieved in each group with reference to the normal weight group. Adjusted for age, smoking habits, coffee consumption, alcohol consumption, reason for infertility, baseline-FSH, total FSH-dose. (B) Number of MII oocytes to median BMI for each BMI group and cycle number. Only ICSI- cycles are displayed. Estimates are shown as back transformed multiple linear regression estimates and each estimate shows the percentage of oocytes retrieved in each group with reference to the normal weight group. Adjusted for age, smoking habits, coffee consumption, alcohol consumption, reason for infertility, baseline-FSH, total FSH-dose.

The median number of oocytes retrieved was comparable in all four BMI groups (Table 1). Fig 2 and Table 2 present the association between BMI and oocyte outcome stratified on cycle number. The overall multiple linear regression analysis on all treatment-cycles, after adjustment for potential confounders, showed no significant difference in the total number of oocytes retrieved per cycle when comparing underweight, overweight and obese with women with normal BMI. The three groups had on average respectively 2% (95% CI: - 10;6), 3% (95%CI: -8;4) and 5% (95% CI: -22;16) fewer oocytes compared to women within the normal range of BMI. However, when stratifying on cycle number, in their first treatment cycle overweight and obese women had significantly fewer oocytes retrieved than women with normal BMI (15% and 17%, respectively, 95% CI: -21;-8 and -26;-6). In the following treatment-cycles (2^nd +^) both overweight and obese approached the average number of oocytes for women with normal BMI, with 4% and 6% more oocytes, respectively. Underweight women had 10% (95% CI: -23;5) fewer oocytes in the first cycle and only 3% (95% CI: -27;28) fewer in the following cycles (2^nd+^) compared to normal-weight women. However, none of these findings were statistically significant (*p* = 0.20 and *p* = 0.87).

**Table 2:** Multiple Linear Regression Model of Oocyte Yield According to BMI and Cycle Number. Each estimate shows the percentage of oocytes retrieved in each group with reference to the normal weight group.

	All treatment-cycles			First treatment-cycle			2^nd+^ treatment-cycle		
BMI group	Crude^a^	Adjusted^a^^,^^b^	p-value	Crude^a^	Adjusted^a^^,^^b^	p-value	Crude^a^	Adjusted^a^^,^^b^	p-value
Underweight	-2 (-21;21)	-5 (-22;16)	0.61	-7 (-21;10)	-10 (-23;5)	0.20	1 (-25;36)	-3 (-72;28)	0.82
Normal	ref	ref		ref	ref		ref	ref	
Overweight	-2 (-7;4)	-3 (-8;4)	0.27	-13 (-19;-6)	-15 (-21;-8)	0.00	5 (-2;13)	4 (-3;12)	0.23
Obese	-1 (-9;7)	-2 (-10;6)	0.45	-15 (-24;-5)	-17 (-26;-7)	0.00	7 (-3;17)	6 (-4;17)	0.27

^a^ Data presented as back transformed estimates (95% confidence interval)

^b^ adjusted for age, smoking habits, coffee consumption, alcohol consumption, reason for infertility, baseline-FSH, total FSH-dose.

### Number of MII Oocytes (only ICSI-cycles)

Underweight and normal weight had on average one more MII oocyte retrieved than overweight and obese women (median 6.0 versus 5.0).

The adjusted analysis for the average number of MII oocytes for all treatment cycles showed that women within the overweight range of BMI had a statistically significantly lower number of MII oocytes harvested compared to women with a normal BMI (*p* = 0.009) (Table 3 and Fig 2). Obese women had 8% (95% CI: -19; 5) fewer MII oocytes compared to normal weight women, but the finding was not statistically significant (*p* = 0.21). Underweight women had 12% (95% CI: -19; 54) more MII oocytes, though not significant (*p* = 0.51). Comparing the number of MII oocytes for the first treatment cycle alone, both overweight and obese women had on average a significantly lower number of MII oocytes (20% and 30% respectively, *p* = 0.003 and *p* = 0.001), than did the normal BMI group. The underweight group was comparable to the normal weight group (2% fewer mature oocytes, *p* = 0.84). In the 2^nd+^ cycles no significant difference was observed in the average number of MII oocytes between underweight, overweight and obese on one hand and normal weight women on the other. In the 2^nd+^ cycles the overweight group though, still had fewer MII oocytes retrieved (9%) than did the normal weight group, but the finding was no longer significant (*p* = 0.12). Both obese and underweight women had more MII oocytes than the normal weight group (19% and 3%) in the 2^nd+^ treatment-cycles. However the finding was not significant.

**Table 3:** Multiple Linear Regression Model of MII Oocyte Yield According to BMI and Cycle Number. Each estimate shows the percentage of MII oocytes retrieved in each group with reference to the normal weight group. Only ICSI-cycles are presented.

	All treatment-cycles			First treatment-cycle			2^nd+^ treatment-cycle		
BMI Group	Crude^a^	Adjusted^a^^,^^b^	p-value	Crude^a^	Adjusted^a^^,^^b^	p-value	Crude^a^	Adjusted^a^^,^^b^	p-value
Underweight	13 (-20;61)	12 (-19;54)	0.51	-3 (-30;33)	-2 (-29;33)	0.84	22 (-21;86)	19 (-19;75)	0.38
Normal	ref	ref		ref	ref		ref	ref	
Overweight	-11 (-20;-2)	-12 (-20;-3)	0.01	-18 (-29;-6)	-20 (-31;-8)	0.00	-8 (-18;4)	-9 (-19;2)	0.12
Obese	-10 (-20;3)	-8 (-19;5)	0.21	-29 (-43;-13)	-30 (-44;-14)	0.00	0 (-14;15)	3 (-11;19)	0.70

^a^ Data presented as back transformed estimates (95% confidence interval)

^b^ adjusted for age, smoking habits, coffee consumption, alcohol consumption, reason for infertility, baseline-FSH, total FSH-dose.

### Total FSH-Dose

As expected, both overweight and obese women did on average receive a higher dose than normal weight women, and the total FSH-dose given increased with increasing BMI (Tables 1 and 4). The adjustment in FSH dose between cycles seemed independent of BMI category, since the increase in FSH-dose from the first to 2^nd+^—cycles was almost similar in all four BMI groups (approximately 400 IU) (Table 4).

**Table 4:** Median Dose of Total FSH (IU) According to BMI and Cycle Number. Stated as median (25/75 percentiles).

	Underweight(< 18,5 kg/m ^2^)	Normal weight (18,5–24,9 5 kg/m ^2^)	Overweight (25,0–29,9 kg/m ^2^)	Obese (≥ 30 kg/m ^2^)
**All cycles**	1572 (1000/1950)	1650 (1200/2025)	1800 (1350/2200)	2000 (1550/2600)
**1**^**st**^ **cycle**	1200 (750/1572)	1350 (1000/1743)	1525 (1050/1950)	1725 (1350/2250)
**2**^**nd+**^ **cycles**	1650 (1200/2200)	1743 (1350/2250)	1875 (1500/2400)	2200 (1650/2700)

### Interaction

The analyses showed no interaction between age and BMI. Adjustment for age alone in the regression analyses (S1 and S2 Tables) did not change the estimates appreciable. When stratifying results by age (≤35 years versus >35 years) the estimates for the two age categories remained essentially the same and were not significantly different from each other, suggesting no effect modification by age (S3 and S4 Tables).There was no interaction between smoking and BMI. Additional adjustment for calendar year did not change the conclusion (S5 and S6 Tables).

### Sensitivity Analysis

A sensitivity analysis was performed, excluding all women with only a first treatment cycle but no subsequent cycles (S7 and S8 Tables). The analysis showed no remarkable difference in the number of oocytes or the number of MII oocytes retrieved compared to the analysis including all first treatment cycle (Tables 2 and 3). Thus, a slightly higher number of MII oocytes were observed in obese women (difference of 11%) compared to the first cycle analysis shown in Table 3. The tendency towards a poorer outcome in first cycles compared to the following cycles was also observed, as was the case for the analysis on the number of MII oocytes.

### Clinical Pregnancy Rate

A post-hoc analysis was performed on clinical pregnancy rate stratified on treatment cycle number. The analysis showed the same trend as observed when analyzing the number of oocytes and number of MII oocytes. Thus, a poorer outcome was observed in first cycles compared to the following cycles for overweight and obese women when compared to normal weight women (Table 5).

**Table 5:** Multiple Logistic Regression Model of Clinical Pregnancy Rate According to BMI and Treatment Cycle Number. A Post- Hoc Analysis. Data presented as OR (95% confidence interval).

	All treatment-cycles			First treatment-cycle			2^nd+^ treatment-cycle		
BMI group	Crude OR	Adjusted OR^a^	p-value	Crude OR	Adjusted OR ^a^	p-value	Crude OR	Adjusted OR^a^	p-value
Underweight	1.12 (0.74;1.71)	0.96 (0.63;1.47)	0.86	1.19 (0.69;2.10)	1.04 (0.58;1.87)	0.89	1.07 (0.64;1.80)	0.90 (0.53;1.53)	0.71
Normal	1.0	1.0		1.0	1.0		1.0	1.0	
Overweight	0.78 (0.65;0.92)	0.77 (0.65;0.91)	0.00	0.64 (0.49;0.83)	0.63 (0.48;0.82)	0.00	0.88 (0.71;1.10)	0.88 (0.71;1.09)	0.24
Obese	0.84 (0.67;1.1)	0.85 (0.67;1.1)	0.16	0.61 (0.41;0.90)	0.62 (0.41;0.93)	0.02	1.01 (0.76;1.35)	1.01 (0.75;1.35)	0.96

^a^ adjusted for age, smoking habits, coffee consumption, alcohol consumption, reason for infertility, baseline-FSH, total FSH-dose.

## Discussion

This study demonstrated a possible interaction or effect modification from cycle number–or a factor related to cycle number- when investigating the effect of BMI on oocyte and MII oocyte outcome in ART. Our findings also indicate that underweight, overweight and obesity do not adversely affect the number of oocytes retrieved in ART when analyzing on all treatment-cycles. No adverse association was found comparing underweight and obesity and the number of MII oocytes (only ICSI-cycles).

To our knowledge, this is the first report to describe a possible interaction related to cycle number when investigating the effect of BMI on oocyte outcome. We wanted to explore whether the effect of BMI on the number of oocytes and number of MII oocytes changed when stratifying the analyses on treatment cycle number. The results showed that overweight and obesity had a significant, negative impact on oocyte number and number of MII in first treatment-cycles, whereas analysing on the following treatment-cycles (2^nd+^) no effect was observed (Tables 2 and 3, Fig 2). This suggests a possible interaction or effect modification related to cycle number.

Analyses regarding the underweight group also revealed more adverse outcomes in first cycles than in the 2^nd+^ for both the total number of oocytes and the total number of MII oocytes, although none of the findings were significant.

So far, the inconsistent findings regarding BMI and oocyte outcome have been described as potentially caused by considerable methodological and clinical heterogeneity in previous studies and lack of confounder adjustment [2,3,16]. Several possible confounders and effect modifiers have been suggested to influence the outcome when investigating BMI and ART, including age, smoking and reason for infertility [3,24,25]. The findings of this report indicate, however, that cycle number may be a possible effect modifier for the association between BMI and oocytes. This could have implications for future studies on BMI and the chance of achieving pregnancy.

The larger volume of distribution in obese women may lead to a requirement for a larger dose for sufficient stimulation than in normal weight women [7,23]. Women with polycystic ovary syndrome (PCO(S)) and obesity may receive insufficient doses of FSH in the first cycle due to mild stimulation in order to prevent ovarian hyperstimulation syndrome. In case of a safe first cycle, increase of the FSH-dose from first cycle to the 2^nd+^, especially in obese women, might explain the observed trend. Insufficient stimulation may result in sub-optimal number of oocytes retrieved, and in that case the dose of FSH may influence the outcome instead of BMI directly. If so, the actual effect of BMI on oocyte outcome should not be assessed by analyzing first treatment cycles only. Estimation of the total dose of FSH given, showed both 1) an increase with increasing BMI and 2) an increase from 1^st^ cycle to subsequent cycles irrespective of BMI. The latter might be explained by the belief or expectation that oocyte yield and pregnancy rates will increase with increasing FSH–dose [26] and could support the theory of FSH having an influence on the outcome. Even so, considering that total FSH-dose increased with increasing BMI and that the total FSH-dose increased in a comparable manner in all BMI groups from the 1st to subsequent cycles, the median dose of total FSH given to the four BMI groups in this study (Tables 1 and 4) could not straight forwardly explain the less optimal oocyte outcome observed in first treatment cycles.

In addition, adjustment for potential small differences in total FSH was performed in the statistical analyses and the differences in oocyte outcome according to cycle number is therefore less likely to be due to suboptimal stimulation among obese women. However, it may be speculated that differences in sensitivity to increasing doses of FSH according to BMI could explain differences in oocyte outcome—both total number and % of MII oocytes—as a response to similar absolute increases in total FSH-dose in 2^nd+^ cycles. It is well known that ovarian tissue damage, for example by ovarian drilling in PCOS women may improve–and even normalize—their endocrinological status [27]. It cannot be excluded that the first oocyte recovery may inflict such damage to the ovarian tissue that changes it towards normal function in overweight and obese women, e.g. a higher number of MII oocytes.

As a consequence of small subgroups of anovulatory women within this dataset, it was not possible to address this issue further without compromising the statistical analyses.

In this study, we also wanted to evaluate the effect of BMI on the number of oocytes and the number of mature oocytes on *all* treatment-cycles. The overall analyses on *all* treatment-cycles showed no significant difference in the number of oocytes retrieved when comparing underweight, overweight and obese with normal weight women, suggesting that BMI, and especially overweight and obesity, has no effect on the number of oocytes. This finding concurs with earlier studies that did not show differences in the number of oocytes retrieved between BMI groups [4,17,19,20,28]. However, the definitions of BMI vary between studies, and a direct comparison with the findings in this study can be difficult. A recent meta-analysis of Rittenberg et al, showed no evidence of high BMI affecting the oocyte outcome, thus supporting the finding in this study [3].

The analyses on the number of MII oocytes on *all* treatment-cycles showed that underweight and obesity did not have any adverse effect. However the overweight–not the obese–did have a significantly lower number of MII oocytes (*p* = 0.009). This finding is somewhat unexpected, as a few studies have reported adverse MII outcome in obese women as well [12,13]. PCOS is the most common cause of anovulatory infertility and has been associated with impaired oocyte maturation [29–31]. In this study, the fraction of anovulatory overweight women (6.7%) and the fraction of anovulatory obese women (10.1%) were higher than the fraction of anovulatory normal weight women (3.3%). This might explain the significantly lower number of MII oocytes observed in overweight women. It is, however, unclear why the same trend was not observed for the obese group as well. The question whether the observed adverse MII outcome in overweight women is truly caused by biological differences among the overweight and normal weight group or perhaps is due to random errors is not clear. A conclusion regarding overweight and number of MII oocytes should therefore be made cautiously.

The results concerning underweight women indicated statistical uncertainties. This was reflected in relatively wide 95% CI, particularly for the MII analysis. Interpretation of the influence of underweight on oocyte outcome in this study should therefore be made with caution.

The strengths of our study were the relatively high number of included cycles in the analysis, and the good confounder adjustment. We included cycles with missing values for some of the explanatory variables to obtain a larger dataset. Imputation was performed, as described in the statistical analysis section, to fill in the missing values, including the variable of total FSH-dose. To exclude a possible bias by including missing values, a sensitivity analysis was performed on cycles with complete data, i.e. without inclusion of missing values (S9 and S10 Tables). The sensitivity analysis showed no remarkable difference in the estimates compared to the results with missing data included. Thus, the observed trend towards differences in outcomes between first cycle and 2^nd+^ cycles also appeared in the analyses on complete cycles. Including missing values is therefore not likely to have influenced the results in this study, and could not explain the results described.

In order to eliminate the possibility of bias from women only having one treatment cycle, a sensitivity analysis was performed, excluding these women (S7 and S8 Tables). Despite a slightly higher number of MII oocytes in obese women in first treatment cycles, the analysis showed no remarkable difference compared to the analysis including all women. The observed trend towards differences in first cycle and 2^nd+^ cycles remained the same. The finding of a higher number of MII oocytes in the obese is most likely due to random error because of lower numbers.

A post-hoc analysis on clinical pregnancy rate showed the same tendency towards a poorer outcome in first treatment cycles for overweight an obese women, suggesting that treatment cycle number not only have an influence when analyzing oocyte number but also on clinical pregnancy rates. However, due to the nature of post hoc analyses the possible influence of treatment cycle number on clinical pregnancy rate when investigating the impact of BMI should be addressed in further studies.

Despite good confounder control and a relative large data set, our study did have its limitations. BMI was assessed at baseline, for which reason it was not possible to eliminate the risk of women gaining or losing weight between cycles. Chavarro et al found that short-term weight loss was associated with a higher yield of MII oocytes, particularly among women who were either overweight or obese at baseline, and who lost 3 kg or more [22]. It can therefore not be ruled out that using baseline BMI rather than cycle specific BMI could have influenced the results on the MII oocyte outcome.

Another possible bias could have occurred by excluding a relative large number of cycles due to lack of information regarding either BMI or treatment type. Even so, it is likely that the information was missing at random and hence would not have influenced the results.

## Conclusion

In conclusion, the findings of this study suggest that investigating the number of oocytes and the number of MII oocytes in first treatment-cycles alone should be carried out cautiously. It was, however, not possible within the settings of this study to explain the observed differences in outcome between the first and the following treatment cycles when stratifying on cycle number. Further studies are warranted to investigate whether there is a biological or medical explanation for these findings. However, these results are noticeable and worthwhile to take into account in future studies investigating the effects of BMI on oocyte and MII outcome and possibly other measures of success or failure in ART, where potential effect modification from cycle number should be taken into account.

## Supporting Information

S1 Table Age Adjusted Multiple Linear Regression Model of Oocyte Yield According to BMI and Cycle Number. (DOCX)

S2 Table Age Adjusted Multiple Linear Regression Model of MII Oocyte Yield According to BMI and Cycle Number. (DOCX)

S3 Table Multiple Linear Regression Model of Oocyte Yield According to BMI and Cycle Number Stratified on Age. (DOCX)

S4 Table Multiple Linear Regression Model of MII Oocyte Yield According to BMI and Cycle Number Stratified on Age. (DOCX)

S5 Table Calendar Adjusted Multiple Linear Regression Model of Oocyte Yield According to BMI and Cycle Number. (DOCX)

S6 Table Calendar Adjusted Multiple Linear Regression Model of MII Oocyte Yield According to BMI and Cycle Number. (DOCX)

S7 Table Sensitivity Analysis of Oocyte Yield. (DOCX)

S8 Table Sensitivity Analysis of MII Oocyte Yield. (DOCX)

S9 Table Sensitivity Analysis of Oocyte Yield with Complete Data. (DOCX)

S10 Table Sensitivity Analysis of MII Oocyte Yield with Complete Data. (DOCX)
