# Supplementary material for: Effect of Female Body Mass Index on Oocyte Quantity in Fertility Treatments (IVF): Treatment Cycle Number Is a Possible Effect Modifier. A Register-Based Cohort Study
Source: PLoS One. 2016 Sep 21;11(9):e0163393. doi: 10.1371/journal.pone.0163393 (PMC5031400; doi:10.1371/journal.pone.0163393)
Supplement: S1 Table — (DOCX) [file pone.0163393.s001.docx]

|  | **All treatment-cycles** | | | **First treatment-cycle** | | | **2^nd+^ treatment-cycle** | | |
| --- | --- | --- | --- | --- | --- | --- | --- | --- | --- |
| **BMI group** | **Crude^a^** | **Adjusted^a,b^** | **p-value** | **Crude^a^** | **Adjusted^a,b^** | **p-value** | **Crude^a^** | **Adjusted^a,b^** | **p-value** |
| Underweight | -2 (-21;21) | -4 (-22;18) | 0.69 | -7 (-21;10) | -8 (-22;9) | 0.35 | 1 (-25;36) | -2 (-27;31) | 0.87 |
| Normal | ref | ref |  | ref | ref |  | ref | ref |  |
| Overweight | -2 (-7;4) | -2 (-8;4) | 0.50 | -13 (-19;-6) | -13 (-19;-6) | 0.00 | 5 (-2;13) | 4 (-3;12) | 0.24 |
| Obese | -1 (-9;7) | -2 (-10;6) | 0.64 | -5 (-24;-5) | -5 (-24;-5) | 0.00 | 7 (-3;17) | 5 (-4;16) | 0.31 |

**S1 Table. Age Adjusted Multiple Linear Regression Model of Oocyte Yield According to BMI and Cycle Number.** Each estimate shows the percentage of oocytes retrieved in each group with reference to the normal weight group.

^a^ Data presented as back transformed estimates (95 % confidence interval) ^b^ adjusted for age
