# Supplementary material for: Effect of Female Body Mass Index on Oocyte Quantity in Fertility Treatments (IVF): Treatment Cycle Number Is a Possible Effect Modifier. A Register-Based Cohort Study
Source: PLoS One. 2016 Sep 21;11(9):e0163393. doi: 10.1371/journal.pone.0163393 (PMC5031400; doi:10.1371/journal.pone.0163393)
Supplement: S2 Table — (DOCX) [file pone.0163393.s002.docx]

**S2 Table. Age adjusted multiple linear regression model of MII oocyte yield according to BMI and cycle number.** Each estimate shows the percentage of MII oocytes retrieved in each group with reference to the normal weight group. Only ICSI cycles are presented.

|  | **All treatment-cycles** | | | **First treatment-cycle** | | | **2^nd+^ treatment-cycle** | | |
| --- | --- | --- | --- | --- | --- | --- | --- | --- | --- |
| **BMI Group** | **Crude^a^** | **Adjusted^a,b^** | **p-value** | **Crude^a^** | **Adjusted^a,b^** | **p-value** | **Crude^a^** | **Adjusted^a,b^** | **p-value** |
| Underweight | 13 (-20;61) | 11 (-22;58) | 0.55 | -3 (-30;33) | -3 (-30;34) | 0.84 | 22 (-21;86) | 17 (-23;79) | 0.75 |
| Normal | ref | ref |  | ref | ref |  | ref | ref |  |
| Overweight | -11 (-20;-2) | -11 (-20;-2) | 0.02 | -18 (-29;-6) | -18 (-29;-6) | 0.01 | -8 (-18;4) | -8 (-19;3) | 0.14 |
| Obese | -10 (-20;3) | -10 (-21;3) | 0.13 | -29 (-43;-13) | -29 (-43;-13) | 0.00 | 0 (-14;15) | -1 (-4;15) | 0.90 |

^a^ Data presented as back transformed estimates (95 % confidence interval) ^b^ adjusted for age
