# Supplementary material for: Effect of Female Body Mass Index on Oocyte Quantity in Fertility Treatments (IVF): Treatment Cycle Number Is a Possible Effect Modifier. A Register-Based Cohort Study
Source: PLoS One. 2016 Sep 21;11(9):e0163393. doi: 10.1371/journal.pone.0163393 (PMC5031400; doi:10.1371/journal.pone.0163393)
Supplement: S3 Table — (DOCX) [file pone.0163393.s003.docx]

**S3 Table. Multiple Linear Regression Model of Oocyte Yield According to BMI and Cycle Number Stratified on Age.** Each estimate shows the percentage of oocytes retrieved in each group with reference to the normal weight group.

|  | **All treatment-cycles** | | **First treatment-cycle** | | **2^nd+^ treatment-cycle** | |
| --- | --- | --- | --- | --- | --- | --- |
| **BMI Group** | **≤ 35 years^a,b^** | **> 35 years^a,b^** | **≤ 35 years^a,b^** | **> 35 years^a,b^** | **≤ 35 years^a,b^** | **> 35 years^a,b^** |
| Underweight | -4 (-23;21) | -8 (-27;17) | -11 (-25;6) | 21 (2;45)^c^ | 1 (-27;40) | -15 (-36;14)^c^ |
| Normal | ref | ref | ref | ref | ref | ref |
| Overweight | -5 (-11;19) | -1 (-14;13) | -17 (-24;-9) | -9 (-26;10) | 5 (-3;13) | 1 (-13;17) |
| Obese | -5 (-14;4) | -2 (-20;19) | -18 (-28;-7) | -13 (-32;11) | 6 (-5;18) | 2 (-19;28) |

^a^ Data presented as back transformed estimates (95 % confidence interval) ^b^ adjusted for smoking habits, coffee consumption, alcohol consumption, reason for infertility, baseline-FSH, total FSH-dose. ^c^ Data affected by a very small number of underweight women > 35 years (n=5)
