# Supplementary material for: Effect of Female Body Mass Index on Oocyte Quantity in Fertility Treatments (IVF): Treatment Cycle Number Is a Possible Effect Modifier. A Register-Based Cohort Study
Source: PLoS One. 2016 Sep 21;11(9):e0163393. doi: 10.1371/journal.pone.0163393 (PMC5031400; doi:10.1371/journal.pone.0163393)
Supplement: S4 Table — (DOCX) [file pone.0163393.s004.docx]

**S4 Table. Multiple Linear Regression Model of MII Oocyte Yield According to BMI and Cycle Number Stratified on Age.** Each estimate shows the percentage of oocytes retrieved in each group with reference to the normal weight group.

|  | **All treatment-cycles** | | **First treatment-cycle** | | **2^nd+^ treatment-cycle** | |
| --- | --- | --- | --- | --- | --- | --- |
| **BMI Group** | **≤ 35 years^a,b^** | **> 35 years^a,b^** | **≤ 35 years^a,b^** | **> 35 years^a,b^** | **≤ 35 years^a,b^** | **> 35 years^a,b^** |
| Underweight | 46 (-18;59) | -9 (0.58;1.44)^c^ | -6 (-31;27) | No data | 26 (-16;88) | 5 (-32;62)^c^ |
| Normal | ref | ref | ref | ref | ref | ref |
| Overweight | -11 (-19;-1) | -15 (-34;9) | -20 (-31;-6) | -21 (48;19) | -6 (-17;6) | -14 (-35;13) |
| Obese | -11 (-24;3) | 6 (19;39) | - 31 (-45;-13) | -25 (-63;3) | 0 (-15;19) | 11 (-13;42) |

^a^ Data presented as back transformed estimates (95 % confidence interval) ^b^ adjusted for smoking habits, coffee consumption, alcohol consumption, reason for infertility, baseline-FSH, total FSH-dose. ^c^ Data may be affected by a very small number of underweight women > 35 years (n=3)
