# Supplementary material for: Effect of Female Body Mass Index on Oocyte Quantity in Fertility Treatments (IVF): Treatment Cycle Number Is a Possible Effect Modifier. A Register-Based Cohort Study
Source: PLoS One. 2016 Sep 21;11(9):e0163393. doi: 10.1371/journal.pone.0163393 (PMC5031400; doi:10.1371/journal.pone.0163393)
Supplement: S5 Table — (DOCX) [file pone.0163393.s005.docx]

|  | **All treatment-cycles** | | **First treatment-cycle** | | **2^nd+^ treatment-cycle** | |
| --- | --- | --- | --- | --- | --- | --- |
| **BMI group** | **Crude^a^** | **Adjusted Calendar year^a^** | **Crude^a^** | **Adjusted Calendar year^a^** | **Crude^a^** | **Adjusted Calendar year^a^** |
| Underweight | -2 (-21;21) | -2 (-21;22) | -7 (-21;10) | -7 (-21;10) | 1 (-25;36) | 1 (-26;37) |
| Normal | 1 (ref) | 1 (ref) | 1 (ref) | 1 (ref) | 1 (ref) | 1 (ref) |
| Overweight | -2 (-7;4) | -2 (-7;4) | -13 (-19;-6) | -13 (-19;-6) | 5 (-2;13) | 5 (-2;13) |
| Obese | -1 (-9;7) | -2 (-9;7) | -15 (-24;-5) | -15 (-21;-5) | 7 (-3;17) | 6 (-4;16) |

**S5 Table. Calendar Year Adjusted Multiple Linear Regression Model of Oocyte Yield According to BMI and Cycle number.** Each estimate shows the percentage of oocytes retrieved in each group with reference to the normal weight group.

^a^ Data presented as back transformed estimates (95 % confidence interval)
