# Supplementary material for: Effect of Female Body Mass Index on Oocyte Quantity in Fertility Treatments (IVF): Treatment Cycle Number Is a Possible Effect Modifier. A Register-Based Cohort Study
Source: PLoS One. 2016 Sep 21;11(9):e0163393. doi: 10.1371/journal.pone.0163393 (PMC5031400; doi:10.1371/journal.pone.0163393)
Supplement: S6 Table — (DOCX) [file pone.0163393.s006.docx]

**S6 Table. Calendar Year Adjusted Multiple Linear Regression Model of MII Oocyte Yield According to BMI and Cycle Number.** Each estimate shows the percentage of MII oocytes retrieved in each group with reference to the normal weight group. Only ICSI-cycles are presented.

|  | **All treatment-cycles** | | **First treatment-cycle** | | **2^nd+^ treatment-cycle** | |
| --- | --- | --- | --- | --- | --- | --- |
| **BMI Group** | **Crude^a^** | **Adjusted Calendar year^a^** | **Crude^a^** | **Adjusted Calendar year^a^** | **Crude^a^** | **Adjusted Calendar year^a^** |
| Underweight | 13 (-20;61) | 13 (-21;60) | -3 (-30;33) | -4 (-30;33) | 22 (-21;86) | 22 (-21;89) |
| Normal | ref | ref | ref | ref | ref | ref |
| Overweight | -11 (-20;-2) | -11 (-20;-2) | -18 (-29;-6) | -19 (-29;-6) | -8 (-18;4) | -8 (-19;3) |
| Obese | -10 (-20;3) | -10 (-21;3) | -29 (-43;-13) | -29 (-43;-13) | 0 (-14;15) | 0 (-14;15) |

^a^ Data presented as back transformed estimates (95 % confidence interval)
