# Supplementary material for: Effect of Female Body Mass Index on Oocyte Quantity in Fertility Treatments (IVF): Treatment Cycle Number Is a Possible Effect Modifier. A Register-Based Cohort Study
Source: PLoS One. 2016 Sep 21;11(9):e0163393. doi: 10.1371/journal.pone.0163393 (PMC5031400; doi:10.1371/journal.pone.0163393)
Supplement: S8 Table — (DOCX) [file pone.0163393.s008.docx]

**S8 Table. Sensitivity Analysis of MII Oocyte Yield.** All women with only 1^st^ treatment cycle are excluded. Multiple linear regression model of MII oocyte and outcome according to BMI and cycle number. Each estimate shows the percentage of MII oocytes retrieved in each group with reference to the normal weight group. Only ICSI cycles are presented.

|  | **All treatment-cycles** | | | **First treatment-cycle** | | | **2^nd+^ treatment-cycle** | | |
| --- | --- | --- | --- | --- | --- | --- | --- | --- | --- |
| **BMI Group** | **Crude^a^** | **Adjusted^a,b^** | **p-value** | **Crude^a^** | **Adjusted^a,b^** | **p-value** | **Crude^a^** | **Adjusted^a,b^** | **p-value** |
| Underweight | 13 (-3;5) | 13 (-22;63) | 0.51 | -9 (-41;43) | -1 (-36;55) | 0.98 | 20 (-23;86) | 17 (-22;74) | 0.45 |
| Normal | ref | ref |  | ref | ref |  | ref | ref |  |
| Overweight | -13 (-22;-3) | -14 (-23;-4) | 0.01 | -29 (-40;-15) | -30 (-42;-17) | 0.00 | -8 (-18;4) | -9 (-19;3) | 0.13 |
| Obese | -5 (-17;9) | -3 (-16;11) | 0.62 | -20 (-37;2) | -19 (-37;3) | 0.08 | 0 (-14;16) | -2 (-12;19) | 0.75 |

^a^ Data presented as back transformed estimates (95 % confidence interval) ^b^ adjusted for age, smoking habits, coffee consumption, alcohol consumption, reason for infertility, baseline-FSH, total FSH-dose
